# The combination of transversus abdominis plane block and rectus sheath block reduced postoperative pain after splenectomy: a randomized trial

**DOI:** 10.1186/s12871-020-0941-1

**Published:** 2020-01-23

**Authors:** Jing-li Zhu, Xue-ting Wang, Jing Gong, Hai-bin Sun, Xiao-qing Zhao, Wei Gao

**Affiliations:** 1 Department of Anesthesiology, the Second Affiliated Hospital of Harbin Medical University, Harbin, Heilongjiang China; 2 Department of Anesthesiology, the Second Affiliated Hospital of Harbin Medical University, Harbin, Heilongjiang China; 3 Department of Anesthesiology, the Second Affiliated Hospital of Harbin Medical University, 246 Xuefu Road, Nangang District, Harbin, Heilongjiang China

**Keywords:** Splenectomy, Transversus abdominis plane block, Rectus sheath block

## Abstract

**Background:**

Splenectomy performed with a curved incision results in severe postoperative pain. The aim of this study was to evaluate the effect of transversus abdominis plane block and rectus sheath block on postoperative pain relief and recovery.

**Methods:**

A total of 150 patients were randomized into the control (C), levobupivacaine (L) and levobupivacaine/morphine (LM) groups. The patients in the C group received only patient-controlled analgesia. The patients in the L and LM groups received transversus abdominis plane block and rectus sheath block with levobupivacaine or levobupivacaine plus morphine. The intraoperative opioid consumption; postoperative pain score; time to first analgesic use; postoperative recovery data, including the times of first exhaust, defecation, oral intake and off-bed activity; the incidence of postoperative nausea and vomiting and antiemetics use; and the satisfaction score were recorded.

**Results:**

Transversus abdominis plane block and rectus sheath block reduced intraoperative opioid consumption. The patients in the LM group showed lower postoperative pain scores, opioid consumption, postoperative nausea and vomiting incidence and antiemetic use and presented shorter recovery times and higher satisfaction scores.

**Conclusions:**

The combination of transversus abdominis plane block and rectus sheath block with levobupivacaine and morphine can improve postoperative pain relief, reduce the consumption of analgesics, and partly accelerate postoperative recovery.

**Trial registration:**

Chinese Clinical Trial Registry, ChiCTR 1,800,015,141, 10 March 2018.

## Background

Splenectomy performed via a curved incision from the subxiphoid region to the anterior axillary line along the left subcostal margin results in injury to muscles, such as the rectus abdominis muscle, the external oblique muscle, the internal oblique muscle and the transversus abdominis muscle, etc. [1]. These broad injuries of the upper abdominal wall are the main contributors to severe postoperative pain [2], resulting in postoperative complications and a prolonged duration of recovery after the operation [3]. Sufficient analgesia could ameliorate postoperative nausea and vomiting (PONV), promote intestinal peristalsis, and enhance the recovery of patients [4]. Although patient-controlled analgesia (PCA) achieves higher patient satisfaction than epidural analgesia, PCA is the less effective of the two [5], while epidural analgesia is contraindicated because of coagulation disorders. Nerve block with the guidance of ultrasound can increase the success, safety and quality of regional nerve blocks [6]. Ultrasound-guided rectus sheath block (RSB) and transversus abdominis plane block (TAPB) were confirmed to reduce postoperative pain and consumption of analgesics, decrease the incidence of postoperative complications and enhance recovery after the operation [7–9]. However, no study has investigated the analgesic efficacy of RSB or TAPB in splenectomy because neither the block range of RSB nor that of TAPB alone is sufficient for the surgical incision.Recently, some studies have applied both RSB and TAPB to reduce postoperative pain [10, 11]. Therefore, in this study, we performed ultrasound-guided RSB and TAPB and investigated their effect on postoperative pain and recovery after splenectomy.

## Methods

### Patients

This prospective, single-centre, randomized, parallel-group, double-blinded trial (Chinese Clinical Trial Registry: ChiCTR 1,800,015,141‍) was approved by the Ethics Committee of Harbin Medical University. The study adhered to the CONSORT guidelines and informed written consent was obtained from all patients.

After institutional review board approval (Harbin Medical University Institutional Research Board: KY2018–003), 150 Chinese patients aged 20–70 years who had an American Society of Anesthesiologists (ASA) Physical Status of II-III and underwent open splenectomy were included in this trial between March 2018 and July 2018. Patients with an ASA Physical Status of IV or higher, an allergy to local anaesthetics, a history of abdominal surgery, a body mass index < 15 kg.m^− 2^ or > 40 kg.m^− 2^ or severe cardiac and/or pulmonary dysfunction were excluded. Patients with acute or chronic preoperative opioid consumption or any other analgesic treatment for chronic pain before surgery, psychiatric or neurological factors (language barrier, neuropsychiatric disorder) were excluded. Patients who required postoperative mechanical ventilation, had sustained excessive haemorrhage (> 1 L of estimated blood loss) or required a massive transfusion and patients with failed nerve block (the needle could not be positioned in the anatomic structure and the drugs failed to enter the interspace) were also excluded.

### Study design

The 150 patients for whom TAPB and RSB were successfully established were randomly divided into 3 groups: a control group (C), a levobupivacaine group (L) and a levobupivacaine/morphine group (LM) (*n* = 50). Patients in group C received general anaesthesia combined with RSB and TAPB with saline, and intravenous PCA for postoperative pain. Patients in groups L and LM received general anaesthesia combined with RSB and TAPB with levobupivacaine 0.2% alone or levobupivacaine 0.2% with morphine 30 μg.kg^− 1^. The dosage of morphine was adjusted according to paravertebral block [12].

All patients were monitored by continuous electrocardiography (ECG) and pulse oximetry (SpO_2_). After local infiltration of lidocaine, the radial artery cannula was inserted to monitor the invasive blood pressure (BP). After induction with 0.03 mg.kg^− 1^ midazolam, 1 mg.kg^− 1^ lidocaine, 0.4 μg.kg^− 1^ sufentanil, 0.5 mg.kg^− 1^ atracurium, and 0.2 mg.kg^− 1^ etomidate, the tracheal intubation was performed. After intubation, the patients were randomized into the groups C, L and LM. On the basis of our clinical experience, the patients in group C received intravenous PCA with sufentanil (0.04 μg.kg^− 1^ h^− 1^) diluted into 150 ml of saline with a PCA device at a rate of 2 ml.h^− 1^ continuously, a 2-ml bolus injection, and PCA with a 15-min lockout interval for postoperative analgesia. Patients in groups L and LM received levobupivacaine (60 ml of levobupivacaine 0.2%) or levobupivacaine combined with morphine (60 ml of levobupivacaine 0.2% and morphine 30 μg.kg^− 1^) for postoperative analgesia. Anaesthesia was maintained with sevoflurane (expiratory concentration 1.5%) and remifentanil. Patients in group C received remifentanil (10 μg.kg^− 1^ h^− 1^), and patients in group L and group LM received remifentanil to maintain their BP and heart rate within the range of 20% from the baseline. If the change in BP and/or heart rate (HR) exceeded 20% of baseline, 1 μg.kg^− 1^ remifentanil or 6 mg ephedrine was injected.

Patients were randomly allocated to group C, L or LM according to a random sequence generated using Stata version 11 software (StataCorp; TX, USA). An independent anaesthesiologist who did not participate in the peri-operative evaluation prepared the drug for each group according to the allocation results. The second anaesthesiologist only performed the nerve block and anaesthesia. Another independent anaesthesiologist who was blinded to the randomization and anaesthesia results only investigated and recorded the peri-operative data.

All patients received RSB and TAPB after intubation in a supine position.

### Ultrasound-guided rectus sheath block

**Fig. 1:**
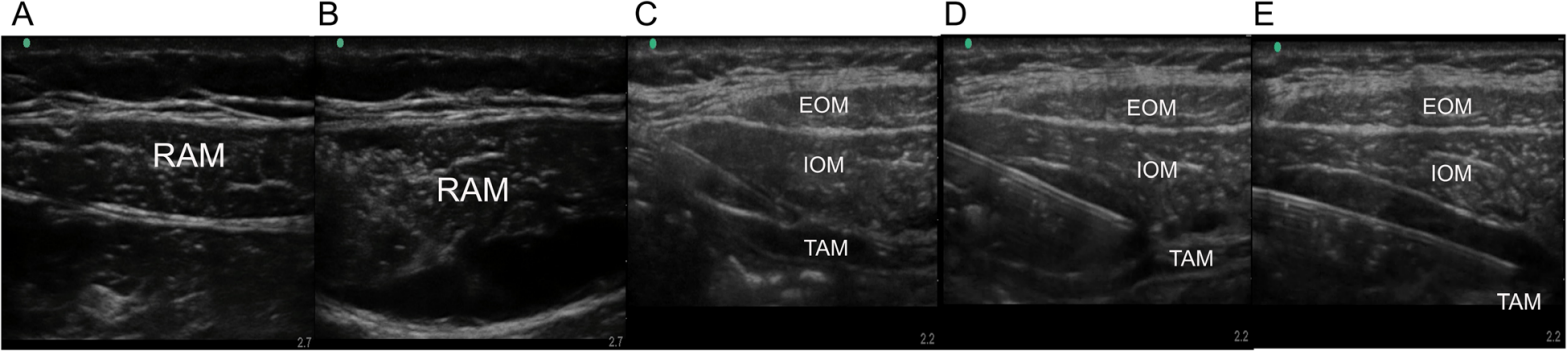
Ultrasound-guided TAPB and RSB External oblique muscle, internal oblique muscle, transversus abdominis muscle, and peritoneum. A and B show ultrasound-guided RSB. The needle tip was positioned in the posterior rectus sheath, and saline was injected. Uniform hydrodissection of the muscle tissue and sheath was critical for the success of RSB, and then, the anaesthetics were injected. *RSB* rectus sheath block, *RAM* rectus abdominis muscle. C, D and E represent ultrasound images of TAPB. The needle tip was positioned in the plane between the internal oblique muscle and the transversus abdominis muscle. After dissection of the plane by injection of saline, the anaesthetics were injected. During the injection of anaesthetics, the needle was advanced further along the transabdominal plane, and the regional anaesthetics were injected step by step to ensure that the entire transabdominal plane was filled with anaesthetics. *TAPB* transversus abdominis plane block, *EOM* external oblique muscle, *IOM* internal oblique muscle, *TAM* transversus abdominis muscle.

We performed RSB at the first and second segments of the rectus abdominis muscle. In brief, under ultrasound guidance (M-Turbo- Ultrasound system; SonoSite, Bothell, WA, USA), a 38-mm broadband linear array ultrasound probe (5–10 MHz) was positioned at the level of the first segment of the subxiphoid region in the transverse plane. The needle was inserted into the skin from the left lateral side to the midline under the middle of the ultrasound probe using an in-plane technique at an angle of approximately 30 degrees to the skin. Under direct vision, we inserted the needle as described above, and after confirmation of the rectus abdominis muscle, we pierced the posterior rectus sheath. Saline was injected to confirm the placement of the needle at the posterior rectus sheath. When the needle placement into the rectus sheath was confirmed, 15 ml of saline, levobupivacaine 0.2% or levobupivacaine 0.2% combined with morphine was injected for the 3 groups after confirmation that no blood was withdrawn, leading to the appearance of a hypoechoic space. Then, RSB of the next segment was performed using the same method and the same volume of anaesthetic solution (Fig. 1a and b).

### Ultrasound-guided transversus abdominis plane block

The ultrasound probe (5–10 MHz) was placed perpendicular to the rectus abdominis muscle and positioned laterally in the left rectus abdominis muscle between the subcostal margin and the iliac crest to obtain the classic view of abdominal layers, including the external oblique muscle, the internal oblique muscle, the transversus abdominis muscle, and the peritoneum. The needle was inserted into the skin from the left rectus abdominis muscle under the middle of the ultrasound probe using an in-plane technique at an angle of approximately 30 degrees to the skin. Under direct vision, after confirmation that no blood was withdrawn, we slowly moved the ultrasound probe from the left rectus abdominis muscle laterally to the left midaxillary line while advancing the needle in transversus abdominis plane. In order to expand the blockade area, when the tip of the needle had been advanced to the beginning of the transabdominal plane, the needle was inserted along the transabdominal plane, and 30 ml of saline, levobupivacaine 0.2% or levobupivacaine 0.2% combined with morphine was injected stepwise as the needle was advanced further; the goal of this technique was to ensure that the whole transabdominal plane was filled with anaesthetics. (Fig. 1c, d and e).

### Procedures and measurements

Blood was collected at completion of the TAPB and then at 10, 20, 30, 60, 90, 120 and 150 min after injection of local anaesthetics so that the concentration of levobupivacaine could be measured using high-performance liquid chromatography (HPLC). Briefly, plasma was collected by centrifugation at 3000 rpm.min^− 1^ for 10 min and kept frozen at − 20 °C for subsequent HPLC (CBM-20A HPLC, Kyoto, Japan) test. The sample flow rate was set to 1.0 ml.min^− 1^, and the detection wavelength was 210 nm. The levobupivacaine concentration was calculated according to the concentration curve of levobupivacaine hydrochloride (Hengrui, Jiangsu, China). The calculated curve of levobupivacaine showed good linearity over a range of 0.5–2000 ng.ml^− 1^ (correlation coefficient ≥ 0.99).

After RSB and TAPB, the right subclavian vein was cannulated to collect blood and infuse blood or fluids, and all patients underwent standard open splenectomy [13]. To avoid the influence of the surgical procedure on postoperative pain, all enrolled patients received RSB and TAPB by the same surgery team. All patients received a left subcostal incision in the supine position. Postoperative analgesia was induced with PCA using sufentanil (0.04 μg.kg^− 1^ h^− 1^) in the C group.

All patients received 40 μg.kg^− 1^ granisetron to prevent PONV [14]. After extubation, all patients were transferred to the post-anaesthesia care unit (PACU). When a patient’s SpO_2_ was over 95% without the use of supplemental oxygen, the patient was transferred to the ward.

Blood loss, infusion (red blood cells [RBCs] and plasma) and consumption of remifentanil were recorded. Postoperative pain at rest and upon coughing was evaluated with a visual analogue scale (VAS) at 0, 2, 4, 6, 24, 48 and 72 h after the operation. The postoperative pain was evaluated by incision and visceral pain (0 = no pain, 10 = worst pain). If the VAS score of any patient was greater than 4, a 3 mg intravenous (i.v.) bolus of morphine was administered, and pain was reassessed after 10–15 min [7]. Other variables were recorded, including time to first exhaust, time to first defecation, time to first oral intake, time to first off-bed activity and incidence of PONV (scored from 0 to 10). Before discharge, all patients scored their satisfaction with postoperative analgesia (poor = 0; fair = 1; good = 2; excellent = 3).

Metoclopramide (10 mg) was intravenously injected if the patients reported a severe episode of nausea (> 7) or any episode of vomiting. The primary outcome was the use of analgesics over 24 h. The pain score, sedation score, satisfaction score postoperative recovery time and PONV were secondary outcomes. To guarantee objective results, the investigator was blinded to the randomization and anaesthesia.

### Sample size

According to our preliminary pilot study and our own experience, the amount of morphine used during the first 72 h after surgery was approximately 15.8 (6.4) mg in patients who received no other analgesics. Approximately 46 patients in each group were required to detect a 25% reduction in morphine between the control and LM groups at 80% power with a two-sided alpha of 0.05.

### Statistical analysis

The normality of the data was analysed with the Shapiro-Wilk test. Normally distributed data are presented as the mean (SD). Non-normally distributed data are presented as the median [interquartile range (IQR)]. Continuous data were analysed with repeated-measures analysis of variance. Normally distributed data were analysed with Student’s t-test, and non-normally distributed data were analysed with the Mann-Whitney U test. Categorical data were analysed with the chi-squared test.

## Results

**Fig. 2:**
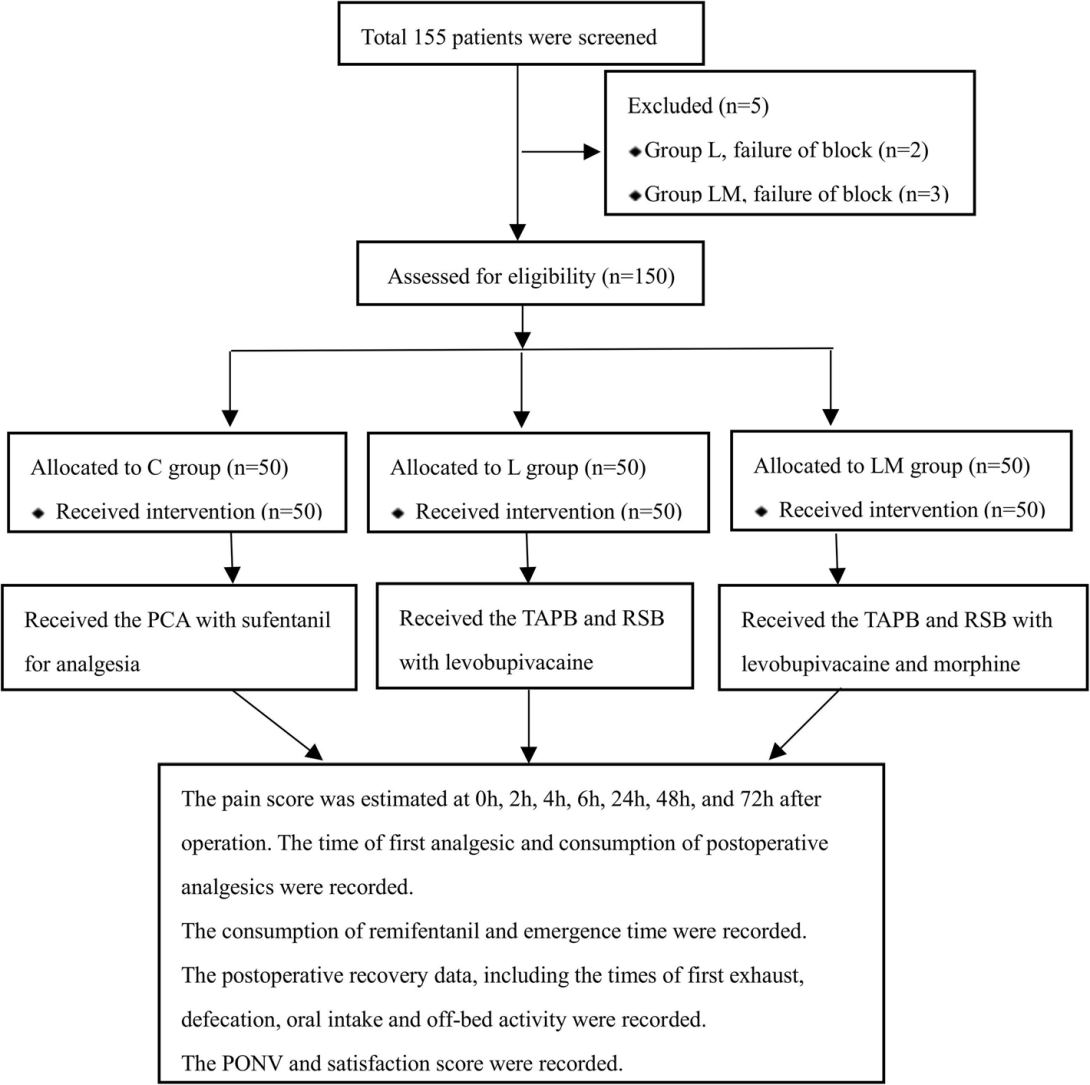
Flow diagram of all patients

Images of TAPB and RSB are shown in Fig. 1. A total of 155 patients were enrolled in this study. Five patients were excluded from the study because of failure of successful block (Fig. 2). There was no difference in demographic characteristics among the 3 groups (Table 1).

**Table 1:** The demographic data of patients in the 3 groups

	C	L	LM	*P* value
Age (year)	54.3 (10.6)	57.1 (10.1)	52.5 (9.3)	0.07
Gender (male n)	26	28	27	0.92
Height (cm)	166.2 (7.8)	164.8 (7.2)	165.3 (8.0)	0.65
Weight (kg)	64.5 (13.7)	64.6 (10.1)	64.5 (11.8)	0.98
Smoking (n)	12	15	17	0.54
Hypertension (n)	14	12	15	0.79
ASA				0.67
II	32	35	36	
III	18	15	14	
HBV	34	36	32	0.69
Hct (%)	36.8 (5.5)	36.9 (6.1)	36.3 (5.5)	0.85
Diagnosis				0.70
Hepatic cirrhosis	32	28	27	
Hypersplenism	11	10	13	
Thrombocytopenic purpura	7	12	10	
Bleeding volume (ml)	349 (192)	318 (192)	332 (133)	0.67
Transfusion volume (ml)	370 (134)	358 (147)	353 (87)	0.78
Operation time (h)	3.0 (1.3)	3.1 (0.9)	3.1 (1.0)	0.86
Anesthesia time (h)	3.8 (1.4)	3.5 (1.0)	3.4 (1.1)	0.21
With pericardial vascular dissection	32	28	27	0.56

Data are expressed as mean (SD) or number

*ASA* the American society of anesthesiologists, *HBV* hepatitis B virus, *Hct* hematocrit

**Fig. 3:**
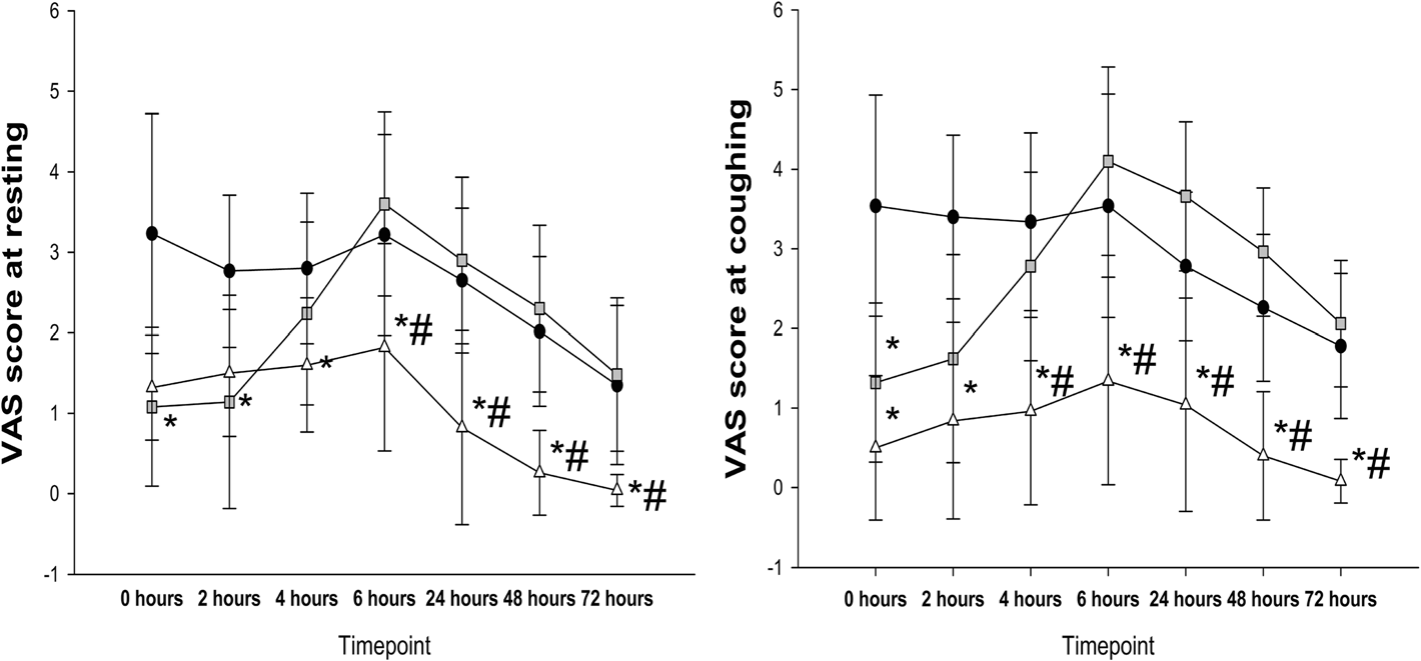
The postoperative pain scores of all patients at rest and during coughing Data are expressed as the mean (SD) for each group (*n* = 50)., and represent the C, L and LM groups, respectively. *, *P* < 0.05 compared with the C group; #, *P* < 0.05 compared with the L group.

The postoperative pain scores at rest in the LM group were significantly lower than those in the C group. The pain scores at rest from 6 to 72 h were significantly lower in the LM group than in the L group. The postoperative pain scores at coughing in the LM group were significantly lower than those in the C group. The pain scores at coughing from 4 to 72 h were significantly lower in the LM group than in the L group (all *P* < 0.05) (Fig. 3). The time to first use of analgesics in the C group was significantly shorter than that in the L and LM groups (*P* < 0.05). The time to first analgesic use in the LM group was longer than that in the L group (*P* < 0.05). The total consumption of morphine in the LM group was less than that in the L group, and the consumption of morphine in the C group was less than that in the L group (*P* < 0.05 for all) (Table 2).

**Table 2:** Comparison of postoperative recovery of patients in the 3 groups

	C	L	LM	P(C vs L)	P(C vs LM)	P(L vs LM)
Remifentanil consumption (mg)	2.97 (0.77)	1.91 (0.73)	1.62 (0.81)	< 0.0001	< 0.0001	0.047
Awaken time (min)	24.04 (6.04)	19.72 (4.83)	18.04 (4.84)	0.0001	< 0.0001	0.083
First analgesic (h)	2.00 (0.50–7.12)	4.65 (2.87–6.82)	13.00 (8.50–17.62)	0.048	< 0.0001	< 0.0001
Total morphine consumption (mg)	12.30 (5.22)	16.68 (5.29)	9.96 (4.51)	< 0.0001	0.0184	< 0.0001
First exhaust (h)	60.4 (14.1)	59.1 (25.4)	54.7 (22.9)	0.76	0.13	0.35
First defecation (h)	85.9 (19.4)	76.1 (23.1)	71.9 (24.1)	0.023	0.0019	0.38
First oral intake (h)	72.6 (13.8)	68.2 (29.1)	65.2 (23.0)	0.32	0.048	0.57
First off-bed (h)	52.8 (22.6)	49.7 (26.6)	46.4 (24.5)	0.53	0.18	0.52
PONV (%)	22%	28%	12%	0.48	0.29	0.04
Metoclopramide (mg)	13.6 (5.1)	15.7 (5.1)	11.4 (3.7)	0.019	0.007	< 0.0001
Satisfaction score	1.8 (0.6)	1.6 (0.6)	2.9 (0.7)	0.51	< 0.0001	< 0.0001

Data are expressed as mean (SD), number (%) or median (IQR)

*PONV* postoperative nausea and vomiting

The intraoperative consumption of remifentanil in the L and LM groups was significantly less than that in the C group (*P* < 0.05). The emergence time in the L and LM groups was significantly shorter than that in the C group (*P* < 0.05), but the difference in emergence time between the L and LM groups was not significant (*P* > 0.05) (Table 2).

Compared with the C group, the times to first exhaust and first off-bed activity time were shortened in the L and LM groups, but the differences among groups were not statistically significant (all *P* > 0.05). The time to first defecation was shorter in the L and LM groups than in the C group (*P* < 0.05), but the difference between the L and LM groups was not statistically significant (*P* > 0.05). The time to oral intake was shorter in the LM group than in the C group (*P* < 0.05) but not the L group (*P* > 0.05). The incidence of PONV and consumption of antiemetic agents were significantly lower in the LM group (*P* < 0.05) than in the C group but not in the L group (*P* > 0.05). The satisfaction score of the LM group was significantly higher than that of the C and L groups (*P* < 0.05). There was no significant difference in satisfaction scores between the C and L groups (*P* > 0.05) (Table 2).

**Fig. 4:**
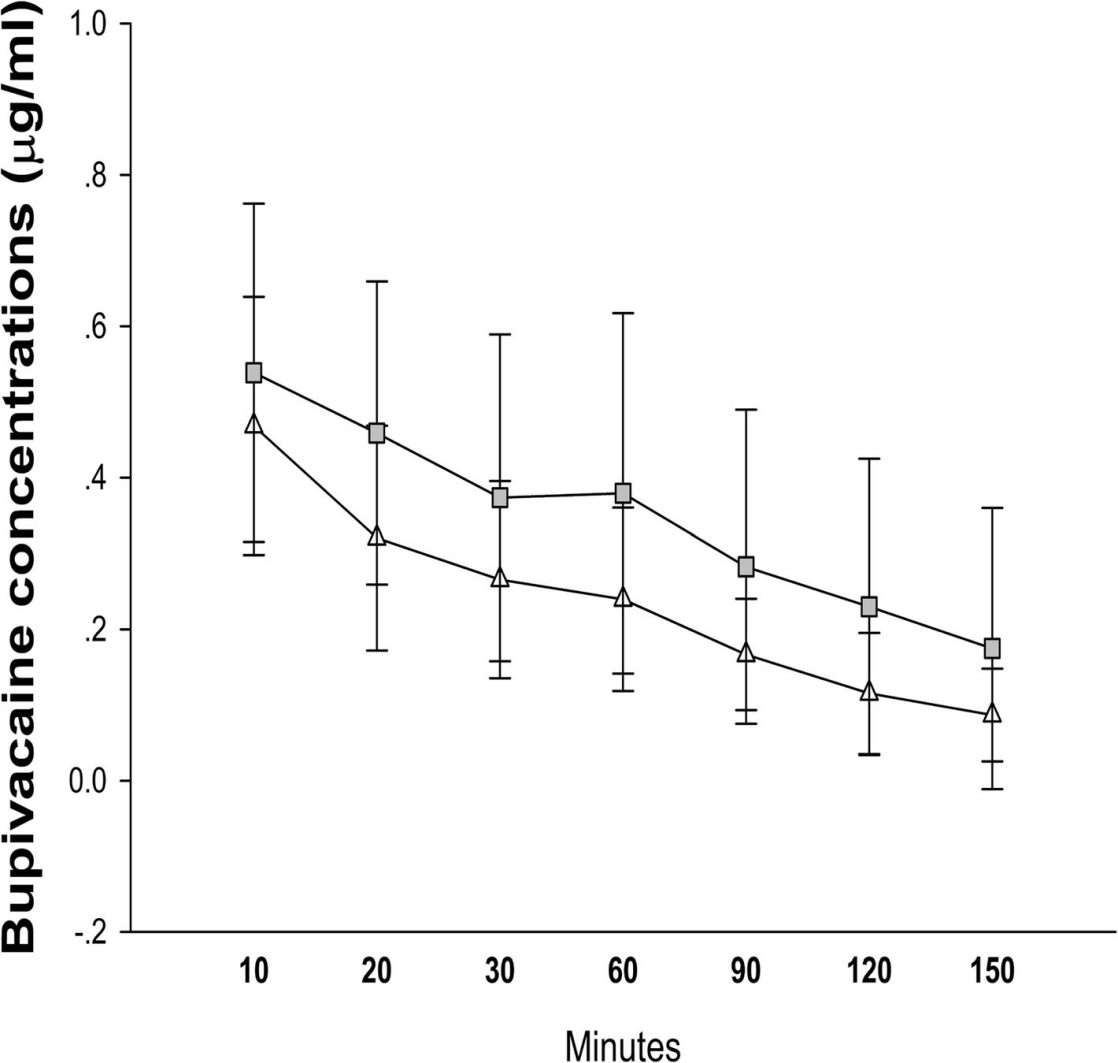
Changes in plasma concentration of levobupivacaine between the L and LM groups. Data are expressed as the mean (SD) for each group (*n* = 50). and represent the L and LM groups, respectively

The levobupivacaine concentrations were significantly different among the groups at 10 min and 30 min after peripheral nerve block. However, no significant difference in the mean maximum plasma concentration of levobupivacaine was observed between the LM group and the L group (Fig. 4). No patient developed clinically severe side effects.

## Discussion

In this study, we found that the combination of RSB and TAPB significantly ameliorated postoperative pain, reduced analgesic consumption, inhibited PONV, and partly promoted postoperative recovery. An enhanced effect of levobupivacaine by concurrent morphine treatment underlies this improvement in outcome.

Postoperative pain can cause a severe stress response [15], respiratory deterioration [16, 17] and neuroendocrine dysfunction [18], preventing early mobilization [17] and even prolonging hospitalization [19]. Moreover, abdominal surgery and consumption of analgesics contribute to PONV [20]. In contrast, sufficient postoperative analgesia is associated with the prevention of postoperative complications and the development of chronic pain, faster postoperative recovery, and a shorter duration of hospitalization [21].

In clinical work, local infiltration and PCA are usually applied in patients with coagulation disorders. However, PCA and local infiltration cannot provide sufficient analgesia [22, 23]. It has been reported that subcostal curve invasion of splenectomy surgery affects multiple nerves and results in severe postoperative pain and the use of many analgesics [13, 24]. Although TAPB can theoretically block the T6-L1 nerves [25], TAPB alone cannot block nerves beyond the costal margins. In our clinical experience, subxiphoid RSB can reduce local somatic pain. Therefore, we hypothesized that the combination of TAPB and RSB could block a more extensive region and provide better postoperative analgesia than either procedure performed alone. Considering its analgesic benefifits in the neuraxial space, we added 30 μg/kg morphine [12]. In this study, we found that TAPB and RSB significantly decreased intraoperative remifentanil consumption. This result was consistent with that of a previous study [26]. There are many afferent nerves that transfer invasive signals from the anterior abdominal wall and lie in potential spaces of the sheath of the rectus abdominis, internal oblique and transversus abdominis muscles. Local anaesthetics injected into these spaces can block the transfer of these invasive signals, further reducing the need for opioids for somatic pain during an operation. Moreover, in this study, we injected anaesthetics step-by-step to fill the entire space of the transversus abdominis plane. In our preliminary study, we found that the combination of TAPB and RSB, with the same method used in this study, can maintain the absence of pain in the entire area of skin of the subcostal curve.

We also found that TAPB and RSB significantly reduced postoperative pain and analgesic consumption and prolonged the time to first analgesia use. Postoperative pain is a serious problem for patients after splenectomy. The curved incision damages many muscles and afferent nerves, contributing to postoperative pain [1, 2]. Postoperative pain not only leads to discomfort and inhibits recovery but also prolongs hospitalization.

The patients underwent splenectomy, which is often combined with coagulation disorders, and were forbidden to receive epidural analgesia. Local infiltration and PCA can provide analgesia for somatic pain, but their efficacy is still debated. TAPB or RSB alone has been shown to provide more effective somatic analgesia for abdominal surgery [7, 11, 27]. However, considering the large range of invasion and the range of blockade from TAPB and RSB alone, we administered a combination of TAPB and RSB to increase the external blockade range. The results suggested that the combination of TAPB and RSB significantly reduced postoperative pain and postoperative analgesic consumption and prolonged the time to first analgesia use. In this study, we found that the VAS score in group C was consistently higher than those in groups L and LM, although the patients in groups L and LM received additional analgesics. In contrast, the patients who received nerve block had lower VAS scores, especially those in the LM group. The time to first analgesic use in the LM group was significantly longer than those in groups C and L. This result may be associated with the metabolic time of levobupivacaine. Without adjuvant drugs, the half-life of levobupivacaine is only approximately 5–7 h, even in muscle spaces. In this study, we added morphine because of its analgesic benefits in the neuraxial space [12]. The synergistic or additive effect of these drugs may prolong the duration of postoperative analgesia [28]. Therefore, the time to first analgesic use in the LM group was significantly longer than that in groups C and L. In this study, the time to first analgesia use in group LM was approximately 14.5 h postoperatively, which was significantly longer than that in groups C and L.

PONV was the most common postoperative complication and was an independent risk factor in postoperative recovery after abdominal surgery. Abdominal surgery and the application of opioids are key risk factors for PONV [20]. In this study, the incidence and severity of PONV were significantly reduced by levobupivacaine plus morphine but not by levobupivacaine alone. This result may be due to the reductions in remifentanil and morphine use. The patients in groups C and L received greater doses of morphine to provide postoperative analgesia than the patients in group LM. Although the LM group also received morphine in local anaesthetics, the absorption of morphine was slow, and the blood concentration of morphine was lower than that in the other two groups. Therefore, morphine had reduced side effects in the LM group. In addition, the inhibition of PONV in the LM group was also attributed to the improvement in postoperative bowel recovery.

In this study, the times to first oral intake, off-bed activity, exhaust and defecation in the LM group were significantly shorter than those in the C and L groups. These results suggest that levobupivacaine plus morphine significantly promoted the postoperative recovery of patients. The promotion of recovery by levobupivacaine plus morphine was not only associated with the effectiveness of the analgesia and the reduction in PONV but also with the reduction in postoperative analgesic use. Due to the effectiveness of analgesia, mobilization can be restored in patients as early as possible [29]. Early mobilization further promotes intestinal peristalsis and early oral intake. Moreover, opioids can lead to bowel dysfunction when they combine with receptors in the bowel wall, thus decreasing bowel peristalsis [30]. In contrast, TAPB had been indicated to promote the postoperative recovery of bowel function [27]. In this study, we found similar results in the LM group but not the L group. The bowel recovery in group L was improved compared with that in group C, but the difference was not statistically significant. We hypothesized that this difference may be due to postoperative opioid consumption. Although the patients received TAPB and RSB, the half-life of levobupivacaine contributed to the short-term analgesia of bupivacaine because the nerve block was performed preoperatively [31, 32]. After the efficacy of levobupivacaine was lost, the patients in group L received more morphine to relieve pain than patients in group C because PCA can continuously provide sufentanil for 75 h.

The novelty of this study is that we developed a new TAPB method to improve the success and efficacy of the procedure. The rate of TAPB failure has been reported to be approximately 10–12% [33, 34], and the efficacy of TAPB remains controversial [35, 36], possibly due to block failure or insufficient block range. In this study, to provide better nerve block efficacy, we administered TAPB via an amended method. We positioned the needle at the beginning of the transverse fascia and then injected the anaesthetics along the path of the advancing needle, proceeding over the entire transverse fascia. With this method, we injected levobupivacaine into the entire transverse fascia, and the success rate was nearly 100%.

Although TAPB and RSB reduced the quantity of opioids needed for postoperative pain relief, the adverse effects of levobupivacaine, including central and cardiac toxicity, must be carefully considered. The systemic toxicity of the anaesthetic is mainly determined by the plasma concentration. It has been indicated that a levobupivacaine plasma concentration exceeding 2620 ng.ml^− 1^ can lead to central nervous system toxicity [37]. In this study, we found that the highest plasma levobupivacaine concentration was less than 800 ng.ml^− 1^, which is significantly lower than the toxic concentration. This result suggests that the dose and injection are safe [38]. Although the application of morphine in nerve block has not been approved, morphine was applied in paravertebral blocks in a previous study [12], and no side effects were reported. In this study, we also did not observe any nerve complications in patients.

## Conclusions

In this study, we found that the combination of TAPB and RSB with levobupivacaine plus morphine significantly reduced postoperative pain and analgesic consumption and promoted postoperative recovery.

## Data Availability

The datasets used and/or analysed during the current study are available from the corresponding author on reasonable request.

## Abbreviations

ASA: American Society of Anesthesiologists
BP: Blood pressure
ECG: Electrocardiography
HPLC: High-performance liquid chromatography
HR: Heart rate
PACU: Post-anaesthesia care unit
PCA: Patient-controlled analgesia
PONV: Postoperative nausea and vomiting
RSB: Rectus sheath block
SpO_2_: Pulse oximetry
TAPB: Transversus abdominis plane block
VAS: Visual analogue scale
